# Computational Approaches to Discover Novel Natural Compounds for SARS‐CoV‐2 Therapeutics

**DOI:** 10.1002/open.202000332

**Published:** 2021-05-19

**Authors:** Shailima Rampogu, Gihwan Lee, Apoorva M. Kulkarni, Donghwan Kim, Sanghwa Yoon, Myeong Ok Kim, Keun Woo Lee

**Affiliations:** ^1^ Division of Life Sciences Research Institute of Natural Science Gyeongsang National University 501 Jinju-daero Jinju 52828 South Korea; ^2^ Division of Life Science and Applied Life Science College of Natural Sciences Gyeongsang National University Jinju South Korea

**Keywords:** SARS-CoV-2, natural compounds, COVID-19, molecular docking, virtual screening, computational studies

## Abstract

Scientists all over the world are facing a challenging task of finding effective therapeutics for the coronavirus disease (COVID‐19). One of the fastest ways of finding putative drug candidates is the use of computational drug discovery approaches. The purpose of the current study is to retrieve natural compounds that have obeyed to drug‐like properties as potential inhibitors. Computational molecular modelling techniques were employed to discover compounds with potential SARS‐CoV‐2 inhibition properties. Accordingly, the InterBioScreen (IBS) database was obtained and was prepared by minimizing the compounds. To the resultant compounds, the absorption, distribution, metabolism, excretion and toxicity (ADMET) and Lipinski's Rule of Five was applied to yield drug‐like compounds. The obtained compounds were subjected to molecular dynamics simulation studies to evaluate their stabilities. In the current article, we have employed the docking based virtual screening method using InterBioScreen (IBS) natural compound database yielding two compounds has potential hits. These compounds have demonstrated higher binding affinity scores than the reference compound together with good pharmacokinetic properties. Additionally, the identified hits have displayed stable interaction results inferred by molecular dynamics simulation results. Taken together, we advocate the use of two natural compounds, **STOCK1N‐71493** and **STOCK1N‐45683** as SARS‐CoV‐2 treatment regime.

## Graphical abstract

**Computational molecular modelling** techniques were employed to discover compounds with potential SARS‐CoV‐2 inhibition properties. The obtained compounds were subjected to molecular dynamics simulation studies to evaluate their stabilities. Additionally, the identified hits have displayed stable interaction results inferred by molecular dynamics simulation results.

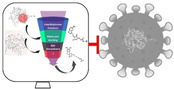

## 1 Introduction

Severe acute respiratory syndrome coronavirus 2 (SARS‐CoV‐2), originated in late December 2019 has spread across the world generating a global pandemic.^[1]^ Currently, the Coronavirus Disease 2019 (COVID‐19) has no effective medication.^[2]^ Coronaviruses (CoV) affect the humans and are grouped into 4 genera, α‐CoV, β‐CoV that are known to infect mammals and γ‐CoV, δ‐CoV predominantly infect birds.^[2]^ Structurally, these viruses are enveloped obtaining the lipid membrane from the host cell and in which the surface proteins of the viruses are encapsulated.^[1]^ These proteins resemble a halo‐like appearance when viewed under the electron microscope. Hence, these group of viruses are called *corona*, meaning garland or crown in Latin.[^1^, ^3^]

The genome of SARS‐CoV‐2 comprises of non‐structural polyprotein open reading frame (ORF)1a/b, which upon proteolytic cleavage generates 15/16 proteins, 4 structural proteins and 5 accessory proteins (ORF3a, ORF6, ORF7, ORF8 and ORF9) with size of 30 kb.[^4^, ^5^, ^6^] The structural proteins are instrumental for SARS‐CoV‐2 assembly and infection. They are the spike (S) surface glycoprotein, the envelope (E) protein, the membrane (M) protein, and the nucleocapsid (N) protein.[^2^, ^7^, ^8^] The infection triggers when Spike protein of the SARS‐CoV‐2 (COVID‐19) interacts with the ACE2 (angiotensin‐converting enzyme 2). For its successful entry into the host cell, the spike protein has to be primed using an enzyme called the TMPRSS2 protease. In brief, the spike protein (virus receptor) interacts with ACE2 (cellular ligand) by TMPRSS2.[^9^, ^10^, ^11^]

After the infection, ORF1a and ORF1b undergo translation and are proteolytically cleaved to form functional proteins.^[12]^ These functional proteins manifest their contribution during viral replication.^[13]^ The non‐structural proteins are nsp1, nsp2, nsp3, nsp4, nsp5, nsp6, nsp7, nsp8, nsp9, nsp10, nsp11, nsp12, nsp13, nsp14, nsp15, nsp16. The nsp1 suppresses host innate immune functions noticed in infected cells which proposes that SARS‐CoV nsp1 may play an important role in SARS‐CoV virulence.^[14]^ Nsp1 protein from SARS‐CoV‐2 interacts with the 40S ribosomal subunit, leading to closure of the mRNA translation in vitro and in cells. Cryo‐electron microscopy guided structural analysis has notified that the Nsp1C terminus interacts to and hinders the mRNA entry tunnel thus blocks RIG−I‐dependent innate immune responses thereby making it a potential target for designing drugs.^[15]^ Studies on nsp2 murine hepatitis virus (MHV) and SARS‐CoV has revealed that nsp2 is dispensable for viral replication, and deletion of coding sequence of nsp2 represses the viral growth and RNA synthesis.^[16]^ Another study recorded that the protein nsp2 interacts with prohibitin 1 (PHB1) and PHB2 two host proteins and may contribute to the intracellular host signalling disturbances amidst SARS‐CoV infections.[^17^, ^18^] Of all the coronavirus proteins, nsp3 is the largest multi domain transmembrane protein.^[19]^ It is generated by pp1a/1ab by the papain‐like protease domain(s), a part of Nsp3,^[20]^ and participates by several roles in the life cycle of the virus by serving as a scaffold protein aiding to bind to itself and to interact with host proteins or other viral nsp's.^[20]^ It is a fundamental requirement for the replication/transcription complex (RTC)^[20]^ formation. Furthermore, nsp3 is involved in the cleaving of the polypeptides, hindering the host innate immune response and stimulating cytokine expression.^[21]^ The nsp4 binds to nsp3 which is a necessity for viral replication and perhaps host protein to bring about the role related to the rearrangement of the membrane in SARS‐CoV.[^8^, ^22^] The nsp5 encodes for M^pro^ and fundamentally cleaves viral polypeptides[^17^, ^19^] and inhibits IFN signalling.^[21]^ The protein nsp6 participates in restricting autophagosome expansion and formation of double‐membrane vesicle (DMV).[^21^, ^23^] The nsp7 cofactors with nsp8 and nsp12, and nsp8 is a cofactor with nsp7 and nsp12.^[21]^ Nsp9 performs dimerization and RNA binding.^[21]^ Additionally, nsp9 is speculated to mediate the replication of virus, cause overall virulence and reproduction of viral genomic RNA.^[24]^ Nsp10 serves as scaffold a protein for nsp14 and nsp16.^[21]^ The nsp12 functions as RNA‐dependent RNA polymerase^[21]^ and its polymerase activity is enhanced in the presence of nsp7 and nsp8 cofactors.^[25]^ The nsp13 performs RNA and DNA duplex‐unwinding.^[26]^ Studies report that nsp13 localizes to membranes, derived from endoplasmic reticulum that are perhaps the sites of RNA synthesis of SARS‐CoV.^[26]^ The nsp14 executes the proofreading of the viral genome^[17]^ and nsp15 has viral endonuclease and chymotrypsin‐like protease activities^[17]^ and the functional role of nsp11 is still unknown.^[21]^ The nsp16 is involved in negative regulation of the innate immunity^[21]^ and shields the RNA of the virus from MDA5 recognition.^[19]^

The RNA molecule of SARS‐CoV‐2 (COVID‐19) is capped at their 5′ end to shield the process of degradation that would be caused by 5′ exoribonucleases, securing efficient translation, and prevents the recognition by the host innate immune system.^[27]^ The non‐structural protein 16 (nsp16) encoding the 2′‐O‐methyltransferase (2′‐O‐MTase) performs the RNA cap modification. It requires the nsp10, which upon its interaction with nsp16 bestows selective 2′‐O‐MTase activity on N7‐methyl guanine RNA caps. This kind of binding mechanism of nsp10 to nsp16 for the accomplishment of the 2′‐O‐MTase activity is a distinctive SARS‐CoV‐2 feature and is not discovered in host cell or other viruses.^[27]^ The binding of nsp10 to nsp16 occurs through ∼930 Å^2^ activation surface in nsp10.^[28]^ This favours nsp16 binding to RNA substrate and the methyl donor S‐adenosyl‐l‐methionine (SAM), thereby leading to the stabilization of SAM‐binding pocket and also elongating the capped RNA‐binding groove.^[27]^ Recent findings have demonstrated three sites for drugs to act upon, such as the SAM binding site, the interface between the nsp16‐nsp10 and the RNA‐binding groove.^[27]^ In the current investigation, we have targeted the SAM‐ binding site for exploiting new leads to combat SARS‐CoV‐2 adapting several computational approaches.

## 2 Results

### 2.1 Binding Affinity Calculations

The molecular docking studies were conducted along with the cocrystallized compound SAM. The dock scores rendered by ‐CDOCKER interaction energy (64.22 kcal/mol) and the GoldScore Fitness (58.42) computed by this compound was designated as an upper limit for the selection of new compounds from the IBS database, as shown in Table 1. Accordingly, two compounds STOCK1N‐45683 and STOCK1N‐71493 have demonstrated a higher to comparable dock score than the reference compounds as listed in Table 1. Additionally, these compounds have shown the key residue interactions with the target protein. In order to delineate on the stabilities of the protein‐ligand complexes, they were upgraded to molecular dynamics simulation (MDS) assessment.

**Table 1:** Binding affinity scores of the new compounds towards the target protein along with the intermolecular interactions.

Name	‐Cdocker Interaction energy [kcal 7mol^−1^]	Goldscore Fitness	Hydrogen Bonds	Alkyl/π Interactions	Van der Waals Interactions
STOCK1N‐45683	64.00	68.99	Asn6841	Tyr6845, Prp6878, Leu6898, Met6929	His6867, Phe6868, Gly6868, Ala6870, Gly6871, Ser6872, Gly6879, Ser6896, Asp6897, Gly6911, Asp6912, Cys6913, Asp6928, Tyr6930, Asp6931, Pro6932, Phe6947
STOCK1N‐71493	62.43	67.62	Gly6871, Cys6913, Asp6928, Met6929	Leu6898	Asn6841, His6867, Phe6868, Gly6869, Ala6870, Ser6872, Pro6878, Gly6879, Ser6896, Asp6897, Asp6912, Cys6913, Tyr6930, Asp6931, Phe6947

#### 2.1.1 Molecular dynamics simulation (MDS) studies

Molecular dynamics simulation (MDS) studies are instrumental in understanding the complex molecular behaviour at the atomistic levels by estimating the experimental results, delineating the molecular motions and biomolecular assemblies.^[29]^ In the current investigation, the MDS was conducted to assess the stability of the protein – ligand complex. The results were interpreted based upon the root mean square deviation (RMSD), binding mode analysis, intermolecular interactions, hydrogen bond analysis and the interaction energy.

### 2.2 Stability and Binding Mode Analysis of STOCK1N‐45683

**Figure 1:**
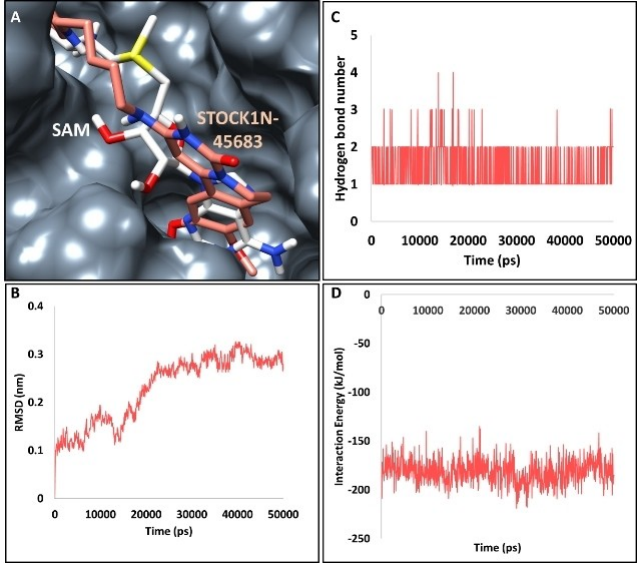
Molecular dynamics simulation results of compound STOCK1N‐45683 at the targets binding pocket. A) Binding mode analysis of STOCK1N‐45683 at the active site. The compound displays a similar binding pattern as that of the cocrystallized compound. B) RMSD guided stability analysis. C) MD inferred number of hydrogen bond interactions. D) Interaction energy between the protein and the ligand during the MD run.

**Figure 2:**
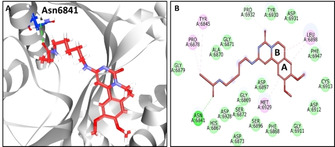
MD inferred intermolecular interactions. A) MD inferred Intermolecular hydrogen bond interactions between the protein and the compound. B) Comprehensive intermolecular interactions.

The compound STOCK1N‐45683 has interacted with the protein target with a dock score of 64 kcal/mol, as shown in Table 1. The MD inferred RMSD results have shown that the protein backbone was stable below 3 nm with an average of 0.22 nm demonstrating that the system was stable without major aberrations, as shown in Figure 1B. Correspondingly, the average structure from last 5 ns was extracted and subsequently superimposed against the X‐ray structure. It was observed that the compound STOCK1N‐45683 has shown a similar binding pattern as that of the SAM held by several key residues, as shown in Figure 1A. This compound has rendered a hydrogen bond interaction with the vital residue Asn6841 noticed with an extended linear chain. The HD21 atom of the Asn6841 and the O17 atom of the ligand have interacted to form a hydrogen bond with a bond length of 2.8 Å, as shown in Figure 2A and Figure 2B. Additionally the residue Leu6898 held the ligand by ring A and ring B prompted by alkyl interaction as displayed in Figure 2B. Ring A also interacted with the Met6929 residue by π‐alkyl interaction. The residues Tyr6845 and Pro6878 have held the ligand via the π‐alkyl interaction accommodating the ligand at the active site of the protein as depicted in Figure 2B. Furthermore, several other residues as illustrated in Figure 2B have aided in clamping the ligand at the binding pocket of the protein as shown in Table 1 and Figure 2B. Additionally the hydrogen bond interactions were monitored throughout the simulations and was noted that the interactions existed during the entire simulations, as shown in Figure 1C defining that the ligand is stably located at the active site of the protein forming stable interactions. Additionally, MD inferred interaction energy between the protein and the ligand was recorded to be ranging between −200 to −150 kJ/mol with an average of −181.58 kJ/mol, as shown in Figure 1D.

### 2.3 Stability and Binding Mode Analysis of STOCK1N‐71493

**Figure 3:**
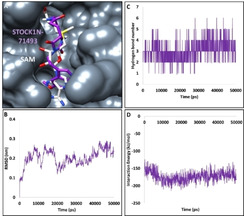
Molecular dynamics simulation results of compound STOCK1N‐71493 at the targets binding pocket. A) Binding mode analysis of STOCK1N‐71493 at the active site. The compound displays a similar binding pattern as that of the cocrystallized compound. B) RMSD guided stability analysis. C) MD inferred number of hydrogen bond interactions. D) Interaction energy between the protein and the ligand during the MD run.

**Figure 4:**
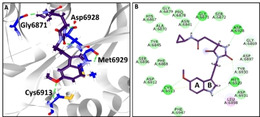
MD inferred intermolecular interactions A) Intermolecular hydrogen bond interactions between the protein and the compound. B) Comprehensive intermolecular interactions.

The compound STOCK1N‐71493 has interacted with the protein demonstrating a dock score of 62.43 kcal/mol, as shown in Table 1, held by several vital residues. The MDS guided RMSD result has displayed that the backbone of the protein was stable below 3 nm, with an average of 0.20 nm, without any major deviations, implying the stability of the protein during the 50 ns simulation run, as shown in Figure 3B. To evaluate the binding mode of the compounds, the last 5 ns structure was retrieved and superimposed against the crystal structure. It was noted that the compound has occupied the binding pocket as that of the cocrystallized compound SAM as depicted in Figure 3A. The compound has formed hydrogen bond interactions with the residues Gly6871, Cys6913, Asp6928 and Met6929, respectively, as shown in Figure 4A. The O atom of Gly6871 has interacted with H36 atom of the ligand with a bond length of 1.8 Å. The HN atom of Cys6913 and the O27 atom of the ligand have interacted with a bond length of 2.7 Å. The OD2 atom of Asp6928 has formed a hydrogen bond with the H29 atom of the ligand rendered by a bond length of 1.7 Å. The residue Met6929 has formed two hydrogen bond interactions. The HN atom of Met6929 and O14 atom of the ligand have generated a hydrogen bond with a length of 2.0 Å. Another hydrogen bond was formed with the O atom of Met6929 and H31 atom of the ligand with a bond length of 2.5 Å, as shown in Figure 4A and 4B. Furthermore, ring A has interacted with Cys6913 by π‐sulphur interaction and with Leu6898 and Met6929 prompted by π‐alkyl interactions. The residue Leu6898 has also interacted with ring B by π‐alkyl interactions as shown in Figure 4B. Furthermore, examining the hydrogen bond interactions have stated that the bonds were consistent throughout the simulations referring to the stable interaction of the ligand, as illustrated in Figure 3C, within the protein at its active site. Furthermore, the interaction energy between the protein and the ligand was recorded to be ranging between −200 to −150 kJ/mol with an average of −173.39 kJ/mol, as shown in Figure 3D.

## 3 Discussion

To find effective therapeutics/drugs against COVID‐19, the current study was performed taking the InterBioScreen natural compounds database for virtual screening that has eventually yielded two compounds as prospective drug candidates. The identified compounds have demonstrated good pharmacokinetic properties as predicted by the *ADMET Descriptors* tool accessible with the DS and have demonstrated favourable drug‐likeness properties, displaying their ability to imbibe with good oral bioavailability as shown Supplementary Table 1. ADMET refers to absorption, distribution, metabolism, excretion and toxicity properties of a compound. A compound qualifying to these parameters could easily escalate during the developmental process. To further ensure their physiochemical properties and synthetic accessibility, the predictions were conducted employing the SwissADME,^[30]^ as displayed in the Supplementary Table 1. These dual predictions have illuminated the usability of the selected compounds against SARS‐CoV‐2.

The two final compounds additionally have demonstrated a good binding affinity score (dock score) in comparison with the reference compound upon using dual molecular docking programmes. Additionally, they have shown stable binding at the proteins active site. The compound STOCKIN‐45683 has shown a stable hydrogen bond interactions with the key residue Asn6841. This interaction was seen with the cocrystallized compound SAM, as shown in Supplementary Figure 1. Furthermore, the residue Leu6898 has rendered an alkyl/π‐alkyl interaction with the compound STOCK1N‐45683. This residue was reported earlier with other ligands as well.^[31]^ These findings elucidate that the identified compound STOCK1N‐45683 could act as potential SARS‐CoV‐2 inhibitor targeting nsp16.

The compound STOCK1N‐71493 has interacted with several residues positioning the ligand at the binding pocket of the target. Herein, we did a meticulous similarity interaction search for the residues that have prompted hydrogen bonds with the compound. The residue Gly6871 has generated a hydrogen bond with the compound STOCK1N‐71493, while it has interacted by a van der Waals interaction with SAM, as shown in Supplementary Figure 1. The residue Cys6913 has displayed a hydrogen bond and π‐sulphur interaction with the compound STOCK1N‐71493. Impressively, the cocrystallized ligand SAM has shown the hydrogen bond interaction as was with the discovered compound. The residue Asp6928 has generated a hydrogen bond interaction with the compound STOCK1N‐71493 similar to SAM compound, as illustrated in Supplementary Figure 1. The residue Met6929 has represented a hydrogen bond interaction with the ligand, while it has established a π‐sulphur and π‐alkyl interaction with SAM as shown in Supplementary Figure 1. This exhaustive analysis guides us to understand that the key residues were preserved by the selected compound enhancing their chances as SARS‐CoV‐2 inhibitors.

A few reports exists on targeting the nsp16, the viral RNA methyltransferase (MTase) for identifying the candidate compounds. Elham Tazikeh‐Lemeski *et al*, have discovered the compounds Raltegravir and Maraviroc as potential inhibitors.^[32]^ In another study, the reseachers have performed the sinefungin (SFG) similarity‐based virtual screening and identified SFG analogue 44601604 as a potential inhibitor.^[33]^ Jiang *et al*. have reported eight compounds that included Hesperidin, Rimegepant, Gs‐9667, and Sonedenoson.^[34]^ The compounds discovered in the current investigation have a varied scaffold from that of the already reported chemical spaces and have projected a satisfactory pharmacokinetic properties providing substantial evidence as plausible inhibitors. To sum up, we suggest the two natural compounds from IBS database as probable SARS‐CoV‐2 inhibitors targeting nsp16.

The identified lipophilicity parameters for both the compounds were in agreement with the limits published in literature such as iLOGP was within −3.93 to 6.46,^[35]^ XLOGP3 was within −0.7 to +5.0,[^36^, ^37^] log P(SILICOS‐IT) was less than 5 (http://silicos‐it.be.s3‐website‐eu‐west‐1.amazonaws.com/software/filter‐it/1.0.2/filter‐it.html#references), WLOGP and MLOGP were less than 2.9 and 1.6,^[38]^ thus indicating that the compounds adhered to drug likeness properties. Computed water solubility parameters such as Log *S* (ESOL) <6,^[30]^ Log *S* (Ali) ≤ 0,^[39]^ Log *S* (SILICOS‐IT) between −4 to −6^[40]^ indicated the compounds to be moderately soluble. The screened compounds have demonstrated acceptable values as mentioned in Supplementary Table 1.

Upon performing the novelty check of the obtained compounds, by using SMILES as an input on PubChem^[41]^ and ChemSpider^[42]^ it was evident that these compounds have not been assessed against SARS‐CoV‐2 or any other disease or target. This finding endorses the novelty of the compounds suggesting that these can be taken up for *in vitro* assessment.

### 3.1 Computational Methods

#### 3.1.1 Protein Selection and Preparation

The target protein for the current study is a non‐structural protein 16 (nsp16) bearing the PDB code: 6W4H, (chain A), which is in complex with nsp10. The nsp16 protein structure is cocrystallized with S‐adenosyl methionine (SAM).^[43]^ The protein was prepared by dislodging the water molecules and the heteroatoms thereafter minimizing the structure. The active site was defined for all the atoms and residues around the cocrystallized compound SAM around 10 Å, as displayed in Figure 1 in the Supporting Information.

#### 3.1.2 Small Molecule Dataset Preparation

**Figure 5:**
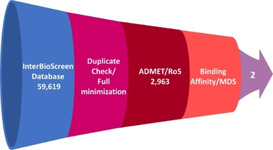
Workflow adapted to identify the candidate compounds.

The small molecule dataset for the current study is taken from the InterBioScreen (IBS) database (https://www.ibscreen.com/). The compounds were downloaded in the .sdf format. These were initially checked for the presence of any duplicates, and were subsequently minimized adapting the *Full Minimization* protocol available with the DS. To the resultant compounds, the ADMET and the Lipinski's Rule of five was applied. The *ADMET Descriptors* tool accessible on the DS was enabled and during this process, the absorption parameters were set at 0 and 1 which represents good and moderate levels. The solubility was defined as 2, 3 and 4 inferring low, good and optimal level and the blood brain barrier level was opted as 2 and 3 defining as medium and low. To the obtained compounds, *Filter by Lipinski* module accessible on the DS was launched to elucidate on the oral bioavailability to mark a drug as effective. Accordingly, the following parameters were assigned, a compound should have no more than 5 hydrogen bond donors, no more than 10 hydrogen bond acceptors, molecular weight no more than 500 and *LogP* no more than 5, which resulted in 2,963 compounds. Additionally, the resultant IBS compounds were subjected to molecular docking studies against the target protein along with the cocrystallized ligand SAM as reference compound. The total process of drug‐like dataset formation and final compound selection is represented in Figure 5.

#### 3.1.3 Molecular docking studies

Molecular docking is one of the superlative methods in which the binding affinities between the target protein and the ligand can be predicted. Additionally, the molecular docking studies elucidate on the probable binding modes of small molecules at the proteins binding pocket. For the current investigation, the CDOCKER programme accessible with the DS was employed.[^44^, ^45^, ^46^, ^47^, ^48^, ^49^, ^50^] The CDOCKER is a CHARMm‐based molecular dynamics (MD) method to dock ligands into a proteins binding site. Correspondingly, random ligand conformations are produced using high‐temperature MD and are subsequently translated into the binding site. Following this, the candidate poses are generated using random rigid‐body rotations followed by simulated annealing. A final minimization is then used to refine the ligand poses. The results are evaluated based upon the ‐CDOCKER interaction energy. To ensure the binding affinity results, a second molecular docking programme, GOLD v5.2.2^[51]^ was additionally employed. GOLD uses a genetic algorithm for docking flexible ligands into receptor binding sites. The results are evaluated based upon the Goldscore Fitness. The binding site is marked around the SAM and the best poses are selected based upon the high /comparable dock score than the reference compound, chosen from the largest cluster, complemented by key residue interactions. The compounds that have obeyed to the aforementioned criteria were subjected to molecular dynamics simulation (MDS) studies.

#### 3.1.4 Molecular Dynamics Simulation (MDS) Studies

The molecular dynamics simulations (MDS) for the selected protein ligand complexes were carried out with CHARMM27 all‐atom force field using GROningen MAchine for Chemical Simulations^[52]^ (GROMACS 2016.16), conducted under periodic boundary conditions. The ligand topologies were generated using the SwissParam program.^[53]^ Inhere, the protein ligand complexes from the CDOCKER were upgraded to MDS. Prior to the simulation, the structures were relaxed by energy minimization to avoid steric clashes or the presence of any inappropriate geometry. A two‐step equilibration was performed after the energy minimization. The first step of the equilibration was conducted under an NVT ensemble (constant number of particles, volume, and temperature) for 1 ns at 300 K using a V‐rescale thermostat. The second step of equilibration was conducted under an NPT (constant number of particles, pressure, and temperature) ensemble for 1 ns, keeping the number of particles, pressure, and temperature at constant. The pressure of the system was monitored at 1 bar using Parrinello‐Rahman barostat.^[54]^ The protein backbone was restrained, while the solvent molecules along with counter‐ions were allowed to move during the equilibration process. The NPT equilibrated structures were escalated to MD simulations for 50 ns. The obtained results were analysed using the GROMACS, visual molecular dynamics (VMD),^[55]^ DS, and Chimera.^[56]^ The LINCS algorithm was employed for the bond constraints^[57]^ and Particle Mesh Ewald (PME) method was utilized to calculate the long‐range electrostatic interactions.^[58]^ The van der Waals interactions were calculated by setting the upper limit of 12 Å.

## 4 Conclusions

The present study is undertaken to swiftly identify drug‐like compounds for SARS‐CoV‐2 infections from the natural compound database namely the IBS database. Our computational studies have determined that the identified compounds have rendered acceptable pharmacokinetic properties together with a binding affinity scores on par with the reference compound. The results inferred from the MD have illuminated the binding potential as inferred by the stable results. Taken together, we advocate the use of two natural compounds, STOCK1N‐45683 and STOCK1N‐71493 into COVID‐19 treatment regime. Additionally, these compounds can also act as starting structures for designing and developing new candidate compounds.

## Abbreviations

COVID‐19: corona virus disease‐2019
SARS‐CoV‐2: Severe acute respiratory syndrome coronavirus 2
CoV: Coronaviruses
ACE2: Angiotensin‐converting enzyme 2
TMPRSS2: Transmembrane protease serine 2
nsp: non‐structural protein
MHV: Murine hepatitis virus
2′‐O‐MTase: 2′‐O‐methyltransferase
MD: molecular dynamics
MDS: molecular dynamics simulation
NVT: constant number of particles volume, and temperature
VMD: visual molecular dynamics
DS: Discovery studio
RMSD: Root mean square deviation.

## Conflict of interest

The authors declare no conflict of interest.

## Supporting information

As a service to our authors and readers, this journal provides supporting information supplied by the authors. Such materials are peer reviewed and may be re‐organized for online delivery, but are not copy‐edited or typeset. Technical support issues arising from supporting information (other than missing files) should be addressed to the authors.

Supplementary
