# Supplementary material for: Computational Approaches to Discover Novel Natural Compounds for SARS‐CoV‐2 Therapeutics
Source: ChemistryOpen. 2021 May 19;10(5):593–9. doi: 10.1002/open.202000332 (PMC8133350; doi:10.1002/open.202000332)
Supplement: Supplementary file 1 — Supplementary [file OPEN-10-593-s001.pdf]

ChemistryOpen

Supporting Information

Computational Approaches to Discover Novel Natural Compounds for SARS-CoV-2 Therapeutics

Shailima Rampogu[+], Gihwan Lee[+], Apoorva M. Kulkarni, Donghwan Kim, Sanghwa Yoon, Myeong Ok Kim,* and Keun Woo Lee*

Supplementary Table 1. Physicochemical Properties and drug-likeness assessment of the identified compounds.

Structure	Physicochemical Properties	
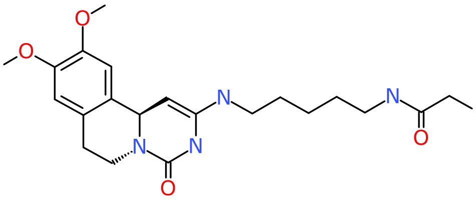 STOCK1N-45683	Formula	C22H32N4O4
	Molecular weight	416.51 g/mol
	Num. heavy atoms	30
	Num. arom. heavy atoms	6
	Fraction Csp3	0.55
	Num. rotatable bonds	11
	Num. H-bond acceptors	4
	Num. H-bond donors	3
	Molar Refractivity	122.26
	TPSA	91.93 Å²
	Lipophilicity	
	Log $P_{o/w}$ (iLOGP)	3.69
	Log $P_{o/w}$ (XLOGP3)	2.28
	Log $P_{o/w}$ (WLOGP)	1.37
	Log $P_{o/w}$ (MLOGP)	1.51
	Log $P_{o/w}$ (SILICOS-IT)	2.35
	Consensus Log $P_{o/w}$	2.24
	Water Solubility	
	Log *S* (ESOL)	-3.28
	Solubility	2.18e-01 mg/ml ; 5.24e-04 mol/l
	Class	Soluble
	Log *S* (Ali)	-3.85
	Solubility	5.91e-02 mg/ml ; 1.42e-04 mol/l
	Class	Soluble
	Log *S* (SILICOS-IT)	-5.89
	Solubility	5.42e-04 mg/ml ; 1.30e-06 mol/l
	Class	Moderately soluble
	Pharmacokinetics	
	GI absorption	High
	BBB permeant	No
	P-gp substrate	Yes
	CYP1A2 inhibitor	No
	CYP2C19 inhibitor	No
	CYP2C9 inhibitor	No
	CYP2D6 inhibitor	Yes
	CYP3A4 inhibitor	Yes

	Log K_p (skin permeation)	-7.22 cm/s
	Druglikeness	
	Lipinski	Yes; 0 violation
	Ghose	Yes
	Veber	No; 1 violation:<br>Rotors>10
	Egan	Yes
	Muegge	Yes
	Bioavailability Score	0.55
	Medicinal Chemistry	
	PAINS	0 alert
	Brenk	0 alert
	Leadlikeness	No; 2 violations:<br>MW>350, Rotors>7
	Synthetic accessibility	4.52
	ADMET (DS)	Solubility 3<br>BBB 3<br>CYP2D6 False<br>Hepatotoxic False<br>PPB False
	Lipinski's Rule (DS)	HBA 8<br>HBD 3<br>Mol_weight 416.514<br>AlogP 2.153<br>Rotatable Bonds 10
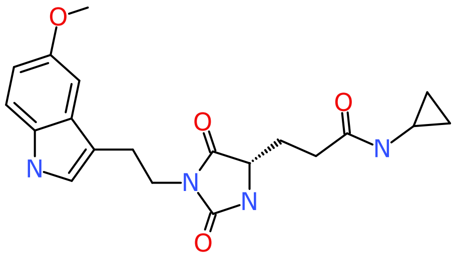<br>STOCK1N-71493	Physicochemical Properties	
	Formula	$C_{20}H_{24}N_4O_4$
	Molecular weight	384.43 g/mol
	Num. heavy atoms	28
	Num. arom. heavy atoms	9
	Fraction Csp3	0.45
	Num. rotatable bonds	9
	Num. H-bond acceptors	4
	Num. H-bond donors	3
	Molar Refractivity	110.92
	TPSA	103.53 Å²
	Lipophilicity	
	Log $P_{o/w}$ (iLOGP)	2.24
	Log $P_{o/w}$ (XLOGP3)	1.39
	Log $P_{o/w}$ (WLOGP)	0.87
	Log $P_{o/w}$ (MLOGP)	0.51
	Log $P_{o/w}$ (SILICOS-IT)	2.48

	Consensus Log $P_{o/w}$	1.50
	Water Solubility	
	Log *S* (ESOL)	-2.74
	Solubility	6.95e-01 mg/ml ; 1.81e-03 mol/l
	Class	Soluble
	Log *S* (Ali)	-3.17
	Solubility	2.61e-01 mg/ml ; 6.80e-04 mol/l
	Class	Soluble
	Log *S* (SILICOS-IT)	-5.33
	Solubility	1.80e-03 mg/ml ; 4.68e-06 mol/l
	Class	Moderately soluble
	Pharmacokinetics	
	GI absorption	High
	BBB permeant	No
	P-gp substrate	Yes
	CYP1A2 inhibitor	No
	CYP2C19 inhibitor	No
	CYP2C9 inhibitor	No
	CYP2D6 inhibitor	No
	CYP3A4 inhibitor	No
	Log K_p (skin permeation)	-7.66 cm/s
	Druglikeness	
	Lipinski	Yes; 0 violation
	Ghose	Yes
	Veber	Yes
	Egan	Yes
	Muegge	Yes
	Bioavailability Score	0.55
	Medicinal Chemistry	
	PAINS	0 alert
	Brenk	1 alert: hydantoin
	Leadlikeness	No; 2 violations: MW>350, Rotors>7
	Synthetic accessibility	3.17
	ADMET (DS)	Solubility 3<br>BBB 3<br>CYP2D6 False<br>Hepatotoxic False<br>PPB False
	Lipinski's Rule (DS)	HBA 8<br>HBD 3<br>Mol_weight 384.429<br>AlogP 1.481

		Rotatable Bonds 8

Supplementary Figure

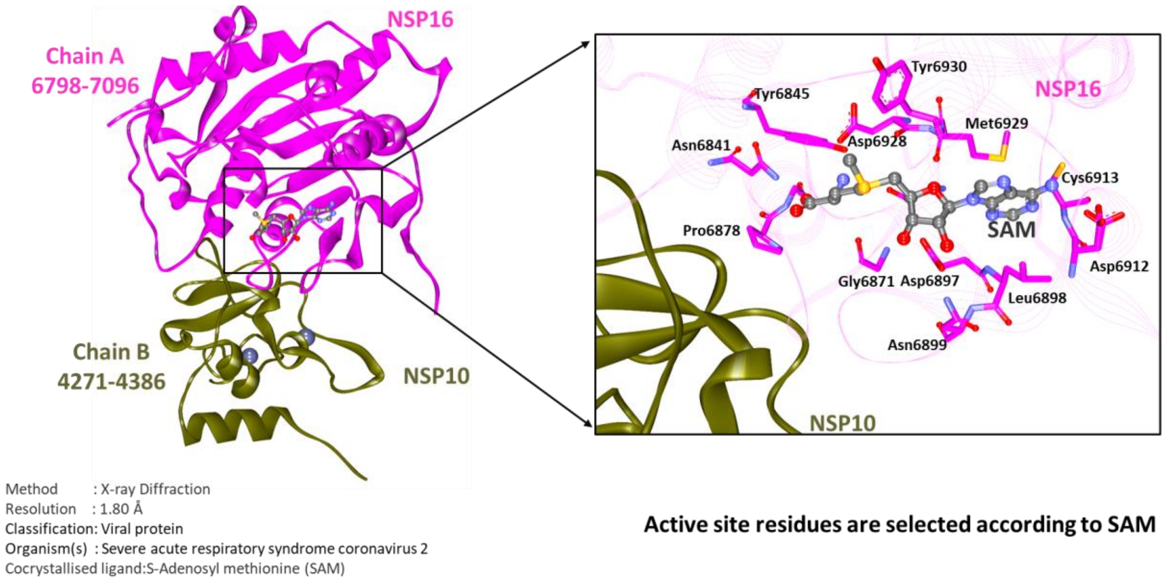

Supplementary Figure 1. Target protein structural details
